# PAR-2 gene expression data and morphology data of rabbit Achilles tenocytes stimulated with PDGF-BB *in vitro*

**DOI:** 10.1016/j.dib.2024.110886

**Published:** 2024-09-02

**Authors:** Gabriella Meier Bürgisser, Olivera Evrova, Dorothea M. Heuberger, Petra Wolint, Julia Rieber, Iris Miescher, Reto A. Schüpbach, Pietro Giovanoli, Maurizio Calcagni, Johanna Buschmann

**Affiliations:** a Division of Plastic Surgery and Hand Surgery, University Hospital Zurich, Sternwartstrasse 14, 8091 Zurich, Switzerland; b Laboratory of Applied Mechanobiology, ETH Zürich, Vladimir-Prelog-Weg 1-5/ 10, 8093 Zurich, Switzerland; c Institute of Intensive Care Medicine, University Hospital Zurich, Sternwartstrasse 14, 8091 Zurich, Switzerland

**Keywords:** rbF2rl1 gene expression, Platelet-derived growth factor-BB, Aspect ratio, Cytoskeleton

## Abstract

The first set of data refers to PAR-2 gene expression with the target gene rbF2rl1 assessed in tenocytes harvested from New Zealand White Rabbits’ Achilles tendons. These tenocytes were stimulated *in vitro* with 20 ng/mL platelet-derived growth factor-BB (PDGF-BB) and compared to the corresponding cell culture without growth factor PDGF-BB. In addition, three inhibitors were tested. In the presence or absence of 40 µM inhibitor concentration and 5 % fetal bovine serum, the following inhibitors were applied: SB203580 = inhibitor for MAPK; LY-294002 = inhibitor for PI3K; PD153035 = inhibitor for EGFR. As control, gene expression was assessed under DMSO = dimethyl sulfoxide (solvent of the inhibitors) or in medium = basal culture medium (with 10 % fetal bovine serum).

The second set of data represents morphological aspects of cytoskeletal reorganization for rabbit Achilles tenocytes stimulated *in vitro* with 20 ng/mL PDGF-BB compared to the corresponding cell culture without PDGF-BB. Data on cell size, on F-actin immunohistochemical labeling intensity, α-tubulin immunohistochemical labeling intensity and on cell aspect ratio (length of the cell divided by its width) are presented. Moreover, analogous to the first set of data, cytoskeletal rearrangement in the presence or absence of the inhibitors SB203580, LY-294002 and PD153035 in the presence or absence of PDGF-BB were assessed.

Specifications Table

**Table:** 

Subject	Genetics; cell biology
Specific subject area	The data were determined for monolayer tenocyte cultures. Tenocytes were harvested from freshly isolated New Zealand White Rabbit Achilles tendons of four rabbits. These monolayer tenocyte cultures were supplemented with the growth factor PDGF-BB, and gene expression for PAR-2 was assessed. Three different inhibitors were used as well, one for MAPK, one for PI3K and finally one for EGFR, respectively. The cell size, F-actin and α-tubulin staining intensity were assessed for ± PDGF-BB conditions; and the aspect ratio of the cells was determined for ± PDGF-BB in the presence or absence of these inhibitors.
Data format	The data consist of raw data and of analysed data (mean and standard deviation of the mean).
Type of data	Excel files of the type *.xlsx* files (data sets with group labels and numbers)
Data collection	**qPCR**: Rabbit Achilles tenocytes were cultured as monolayers and RNA was extracted by an **RNeasy Plus Mini Kit** from Qiagen, Hilden, Germany; followed by reverse transcription with the **iScript Advanced cDNA Synthesis Kit** (Bio-Rad, Cressier, Switzerland). The quantitative real-time PCR reactions were performed with C**FX Connect Real-Time PCR** Detection System (Bio-Rad, Cressier, Switzerland) and **SsoAdvanced SYBR Green Supermix** (Bio-Rad, Cressier, Switzerland). **Microscope**: As for the immunhistochemically labeled cells we used a microscope to analyze the cell size and morphology: **Leica Microscope 6000 B**, Leica Camera AG, Germany; with laser405 for DAPI; laser488 for Phalloidin and laser633 for Alexa647.
Data source location	All data were collected in Zurich, Switzerland, at the University Hospital Zurich.
Data accessibility	**Repository name**: Mendeley Data **Data identification number**: 10.17632/mk7fjwhcpf.1 and 10.17632/55fk93mssn.1 **Direct URL** to data: https://data.mendeley.com/datasets/mk7fjwhcpf/1 https://data.mendeley.com/datasets/55fk93mssn/1
Related research article	Electrospun tube reduces adhesion in rabbit Achilles tendon 12 weeks post-surgery without PAR-2 overexpression - PubMed (nih.gov)

## 1 Value of the Data

• These data can be reused, if other cell types than tenocytes are stimulated with PDGF-BB *in vitro* – for a direct comparison of their PAR-2 gene expression and morphological features.
• These data can be reused, if tenocytes from other species than rabbits are stimulated with PDGF-BB *in vitro* to be compared to our data determined for rabbit tenocytes.
• These data can be reused for experimental comparison of rabbit Achilles tenocyte stimulation with other growth factors than PDGF-BB, if PAR-2 gene expression and/or morphology of the cells is investigated.
• Our data may help researchers in elucidating signalling mechanisms relating to angiogenesis, inflammation, or other pathways, where PDGF-BB plays a regulatory role as a growth factor; particularly of interest to other researchers may the data assessed under several different inhibitors, such as inhibitor for MAPK, PI3K and EGFR, respectively.

## 2 Background

As surgical tendon repair suffers from two main problems, that is adhesion formation to the surrounding tissue and re-rupture caused by inferior mechanical properties after healing [1], the design of novel implant materials aiding tendon rupture repair has envisioned incorporated growth factors to be slowly released to the wound site. Among them, PDGF-BB with its chemotactic and pro-angiogenic properties [2] has been found to enhance the ultimate tensile strength of fully transected rabbit Achilles tendons three weeks post-operation by a factor of two in comparison with tendons treated with the same implant but without PDGF-BB[3]. As PAR-2 protein plays a pivotal role in inflammatory reactions [4], our original motivation was to find out how PDGF-BB supplementation to rabbit Achilles tenocyte cultures influences PAR-2 gene expression (target gene for PAR-2 protein is *rbF2rl1*). In addition, we determined the size of the tenocytes as well as their aspect ratio in order to assess eventual cytoskeletal reorganization under PDGF-BB supplementation. A further aspect was to use several inhibitors to get at least a partial insight into mechanisms responsible for the effects observed under PDGF-BB compared to cell cultures without PDGF-BB.

## 3 Data Description

The data are stored as Microsoft Excel (Microsoft Corporation, Redmond, WA, USA) files (.xlsx files) in the Mendeley Data repository service PAR-2 gene expression in PDGF-BB stimulated rabbit Achilles tenocytes *in vitro* - Mendeley Data and Cytoskeletal reorganization of rabbit Achilles tenocytes stimulated with PDGF-BB *in vitro* - Mendeley Data.

The first repository PAR-2 gene expression in PDGF-BB stimulated rabbit Achilles tenocytes *in vitro* - Mendeley Data includes **two** excel files, named qPCR 4A_xlsx and qPCR 4B_xlsx.

1. **qPCR data for rbF2rl1 gene expression under PDGF-BB supplementation to tenocytes**

**File 1** qPCR 4A_.xlsx: sheet *2 - Results_PW*

This excel file is open access published in Mendeley Data PAR-2 gene expression in PDGF-BB stimulated rabbit Achilles tenocytes *in vitro* - Mendeley Data and contains the sample name in column B, the sample number in column C, the run date in column D, the number of the plate that was run in column E, the name of the reference gene in column F, the CT value of the reference gene in column G, the CT mean values of the reference gene (18S) in column H, the name of the target gene in column I, the CT value of the target gene in column J, the calculated dCT values in column K, the mean of the calculated dCT values in column L, the calculated ddCT values in column M, the calculated 2^-ddCT values in column N, the mean of the calculated 2^-ddCT values in column O and the standard deviation of the calculated 2^-ddCT values in column P. Furthermore, columns R-V represent the summary of all output data, with column R denoting the time point and rabbit number (for example 6 h - R11 = 6 h after stimulation and tenocytes extracted from rabbit 11; or d3 – R7 = day 3 after stimulation and tenocytes from rabbit 7), with column S showing mean values without PDGF-BB (w/o PDGF), column T denoting mean values in the presence of PDGF-BB (with PDGF), in column U showing standard deviation of the mean values without PDGF-BB and finally column V denoting standard deviation of the mean values with PDGF-BB.

**File 2** qPCR 4B_.xlsx: sheet *20211030 - Results_IM*

This excel file is open access published in Mendeley Data PAR-2 gene expression in PDGF-BB stimulated rabbit Achilles tenocytes *in vitro* - Mendeley Data and contains the sample name in column B, the sample number in column C, the run date in column D, the number of the plate that was run in column E, the name of the reference gene in column F, the CT value of the reference gene in column G, the CT mean values of the reference gene (18S) in column H, the name of the target gene in column I, the CT value of the target gene in column J, the calculated dCT values in column K, the mean of the calculated dCT values in column L, the calculated ddCT values in column M, the calculated 2^-ddCT values in column N, the mean of the calculated 2^-ddCT values in column O and the standard deviation of the calculated 2^-ddCT values in column P. Furthermore, columns R-T represent the summary of the output data, with column R denoting the time point, rabbit number and inhibitor as well as growth factor application (for example d3, R7, + SB203580, + PDGF-BB = day 3 after stimulation, tenocytes extracted from rabbit 7 in the presence of the inhibitor SB203580 and in the presence of PDGF-BB), with column S showing mean values and column T denoting standard deviation of the mean values.

2. **Cell morphology and cytoskeletal reorganization under PDGF-BB supplementation to tenocytes**

The second repository called Cytoskeletal reorganization of rabbit Achilles tenocytes stimulated with PDGF-BB *in vitro* - Mendeley Data contains **one** Excel file named *Morphology.xlsx*.

**File** Morphology.xlsx with four sheets

The file Morphology.xlsx has four sheets, named *5C Size, 5D F actin, 5E a-tubulin* and *5F aspect ratio*, respectively. In the sheet *5C Size*, the size of the cells in micrometers is presented in column A, while in column B the group is shown, either w/o meaning without PDGF-BB supplementation to the cells or with denoting that there was a supplementation of PDGF-BB to the cell culture. In the sheet *5D F actin*, the F actin green intensity is given of the cells in column A, while in column B the group is shown, either w/o meaning without PDGF-BB supplementation to the cells or with denoting that there was a supplementation of PDGF-BB to the cell culture. In the sheet *5E a-tubulin*, the alpha-tubulin red intensity of the cells is presented in column A, while in column B the group is shown, either w/o meaning without PDGF-BB supplementation to the cells or with denoting that there was a supplementation of PDGF-BB to the cell culture. In the sheet *5F aspect ratio*, column A represents the group name, B the length of the cells in micrometre, C the width of the cells in micrometre, D the calculated aspect ratio, E the well number in the plate, F the PDGF-BB supplementation status (either with or without PDGF-BB), G the calculated mean of the values for a specific experimental group whose name is presented in column I, and finally column H with the standard deviation of the mean of the values for a specific experimental group whose name is presented in column I.

## 4 Experimental Design, Materials and Methods

### 4.1 In vitro rabbit Achilles tenocyte culture

Rabbit tenocytes were harvested from Achilles tendons of female New Zealand White rabbits aged 12 to 16 weeks utilizing the cell migration method (Veterinary license of Canton Zurich, reference number 255/15). For this aim, tendons harvested from the rabbits and they were washed with Hank's Balanced Salt Solution (1× HBSS with Ca^2+^ and Mg^2+^, Thermo Fisher Scientific, Rockford, IL, USA) with 200 µg/mL gentamycin (Biowest, Nuaillé, France) as well as 2.5 µg/mL amphotericin B (Biowest, Nuaillé, France). After cleaning them from the adjacent tissue, the middle area of the tendons was cut into mini pieces (< 2 mm) and washed three times with 1× HBSS buffer. Afterwards, several tissue pieces were transferred into each tissue culture plate (PrimariaTM, Corning, New York, NY, USA) and a drop of cell culture medium (Ham's F12 (Biowest, Nuaillé, France), 10 % FBS (Biowest, Nuaillé, France), 200 µg/mL gentamycin, and 2.5 µg/mL amphotericin B) was added onto each tissue piece. Tissues attached on the cell culture plates for 2 h at 37 °C and 5 % CO_2,_ before 10 mL of cell culture medium were added to each plate.

During the first five days, the plates with the tissues were not moved in order to minimize and avoid tissue detachment upon plate movement and to allow cells to start migrating out from these tissues. The first medium change was done after five days. From then onwards, the culture medium was changed every third day. After two weeks, tissue pieces were removed from the plates. For another week, cells were allowed to proliferate before they were cryopreserved. Cryopreserved rabbit tenocytes were thawed, resuspended in culture medium (Ham's F12 with 10 % FBS and 50 µg/mL gentamycin) and cultured at 37 °C and 5 % CO_2_. The culture medium was changed every second day. Only tenocytes between passages 2 and 4 (P2–P4) were used in all *in vitro* cell culture experiments, because at higher passages there might be a phenotypic drift typical for tendon cells [5].

Cells were stained for F-actin cytoskeleton (Phalloidin-Alexa488) and for α-tubulin (Anti-alpha tubulin antibody [TU-01] (ab7750)) when 80 % confluency was reached after one week of cell culture in Ham's F12 culture medium with 10 % FBS and 50 µg/mL gentamycin. Specifically, cells were washed with PBS and then fixed with formaldehyde (4 %) for 15 min at room temperature. Finally, after removal of the formaldehyde, the cells were rinsed twice with PBS.

Permeabilisation of the cells was performed with 0.5 % Triton-X 100 in PBS for 20 min. Afterwards, the solution was aspirated and the cells were rinsed again twice with PBS. Subsequently, cells were incubated with the primary antibody for α-tubulin (diluted 1:200, final concentration 5 µg/mL) for 12 h at 4 °C. Then, cells were incubated with an antibody/buffer suspension, composed of the second antibody (Alexa647-anti mouse (A21238), final concentration 10 µg/mL), phalloidin (20 µg/mL) and DAPI (4′6-diamidino-2-phenylindole di-lactate, Sigma-Aldrich, final concentration 5 µg/mL) in PBS, at a pH 7.4, for 1 h at room temperature, under light protection. Samples were rinsed three times for five minutes with PBS before analysis with a microscope (Leica Microscope 6000 B, Leica Camera AG, Germany; with laser405 for DAPI; laser488 for Phalloidin and laser633 for Alexa647). Using Fiji Image J 1.53a software, all images were analyzed for size of the cells (i.e. length, *n* = 35 cells), their aspect ratio (n = 35), F-actin green intensity (*n* = 30 Fields of View, FOVs) and α-tubulin red intensity (*n* = 30 FOVs), respectively, utilizing histograms of the corresponding FOVs.

### 4.2 Real-time PCR

Rabbit tenocytes were isolated and cultured as described above and seeded into 12-well plates (TPP, Trasadingen, Switzerland, growth area per well: 3.60 cm^2^) with a starting cell density of 40,000 cells per well. Cells were allowed to attach during the night before the medium was changed to medium with PDGF-BB (20 ng/mL) or to control medium without any supplementation (Ham's F12, 10 % FBS, 50 µg/mL gentamycin). PDGF- BB in a concentration of 20 ng/mL was added freshly to the medium during every medium exchange. The medium was changed every second day, and cells were stimulated with PDGF-BB for a total of two weeks. After 3, 7 and 14 days samples were collected and analyzed. Rabbit tenocytes from four different animals were used (*n* = 4 donors) and experiments were performed in triplicates for each donor.

At day 3, 7 or 14, respectively, total RNA was isolated using the RNeasy Plus Mini Kit (Qiagen, Hilden, Germany, Switzerland) with RNase-free DNase treatment (Qiagen, Hilden, Germany), following the manufacturer's protocol. For reverse transcription (RT), 500 ng of total RNA was reverse transcribed into cDNA in a reaction volume of 20 µL using the iScript Advanced cDNA Synthesis Kit (Bio-Rad, Cressier, Switzerland). Real-Time PCR reactions were performed on the corresponding cDNA samples (5 ng cDNA per reaction), utilizing the CFX Connect Real-Time PCR Detection System (Bio-Rad, Cressier, Switzerland) and SsoAdvanced SYBR Green Supermix (Bio-Rad, Cressier, Switzerland). The PCR reactions were performed at 95 °C for 3 min, followed by 39 cycles of 95 °C for 10 s and 62 °C for 30 s. Technical duplicates were performed for each sample. The primer sequences for PAR-2 forward were (5′−3′): TTC CCG GCC TTC CTC ACG G and reverse CTT CTG GGA GCT GAG GGA C. The primers were received from Microsynth, Balgach, Switzerland. Relative expression analysis was performed using the comparative 2^−ΔΔCT^ method, having gapdH as reference gene, being stable over the two conditions analyzed. Results are presented as fold change normalized to control, compared to samples cultivated without PDGF-BB: The condition without PDGF-BB was set to 1.

## Limitations

The experiments with PDGF-BB supplementation were only performed at a concentration of 20 ng/mL PDGF-BB, and the determined data are therefore limited only to this concentration. No lower or higher concentrations were investigated.

## Ethics Statement

The rabbit Achilles tenocytes were isolated from rabbits with the animal license approved by the veterinary office of Canton Zurich, Switzerland, reference number 255/15.

## CRediT Author Statement

**Gabriella Meier Bürgisser**: Conceptualization, Methodology, Data curation, Investigation, Writing – Original draft preparation, Writing – Reviewing and Editing. **Olivera Evrova**: Data curation, Writing – editing. **Dorothea M. Heuberger**: Data curation, Writing – Reviewing and Editing. **Petra Wolint**: Data curation, Writing – Reviewing and Editing. **Julia Rieber**: Data curation, Writing – Reviewing and Editing. **Iris Miescher**: Data curation, Writing – Reviewing and Editing. **Reto A. Schüpbach**: Supervision, Writing – Reviewing and Editing. **Pietro Giovanoli**: Supervision, Writing – Reviewing and Editing. **Maurizio Calcagni**: Supervision, Writing – Reviewing and Editing. **Johanna Buschmann**: Conceptualization, Supervision, Methodology, Writing - Original draft preparation, Writing – Reviewing and Editing, Fund raising.

## Data Availability

Cytoskeletal reorganization of rabbit Achilles tenocytes stimulated with PDGF-BB in vitro (Original data) (Mendeley Data). PAR-2 gene expression in PDGF-BB stimulated rabbit Achilles tenocytes in vitro (Original data) (Mendeley Data).
